# Study protocol: seeking treatment for gambling – a 10-year retrospective clinical and register study of gambling patterns and physical and psychiatric comorbidity in patients with gambling disorder

**DOI:** 10.1186/s12954-026-01565-0

**Published:** 2026-10-16

**Authors:** Anders Håkansson, Yassir Abdul Rahim, Molly Miles, Carolina Widinghoff

**Affiliations:** 1 Malmö Addiction Center, Department of Clinical Sciences Lund, Psychiatry, Lund University, S Förstadsgatan 35, Fourth Floor, Malmö, S-205 02 Sweden; 2 Malmö Addiction Center, Skåne University Hospital, Psychiatry, Malmö, Sweden; 3 Child and Adolescent Psychiatry, Skåne University Hospital, Malmö, Sweden

**Keywords:** Gambling disorder, Comorbidity, Mental health disorders, Psychiatric disorders, Somatic disorders, Behavioral addiction, Self-exclusion

## Abstract

**Background:**

Gambling disorder, the first non-substance-related addictive disorder to be recognized, can have a severe impact on health and psycho-social well-being, but treatment seeking is often low, and the documentation of clinical needs and medical comorbidities is sparser than for substance use disorders. The present study protocol describes a large clinical and register study which aims to describe physical and psychiatric comorbidities in patients seeking treatment at a regional gambling disorder treatment unit within addiction health care, and how comorbidity, routes of referral, and psychiatric medications are associated with problem gambling patterns, self-exclusion from gambling, gambling despite self-exclusion, gender, age, psycho-social living conditions, and over-indebtedness. In addition, the study aims to map out prior contacts of care in treatment-seeking patients, aiming to lay the ground for improved and earlier detection of gambling problems in diverse clinical settings.

**Methods:**

Health care documentation will be studied for all patients seeking treatment for gambling-related problems (estimated N around 1,200) for the entire 10-year period since the opening of a specialized treatment unit in the Skåne region, Sweden, in December, 2015, through November, 2025. In addition to data from health care records, treatment episode data will be run against a regional health care register describing all other primary care and specialized care contacts in the region, including out-patient and in-patient episodes and diagnoses, for both physical and mental health disorders.

**Discussion:**

The present project can describe – over time – the full range of socio-demographic conditions and comorbidities, not only regarding mental health but also physical disorders, in clinical patients with gambling disorder. Results can therefore guide efforts for detection of gambling problems in other types of social service or health care settings. User perspectives are ensured through the collaboration with a patient and peer support organization, and public implementation is ensured through continuous collaboration with political decision makers and coordinating leading stakeholders in social services and health care in the region and elsewhere.

**Trial registration:**

ClinicalTrials.gov, Clinical Trials identifier number NCT07488871, date of registration March 9, 2026.

## Background

Gambling for money can cause severe financial problems and may severely impact mental health and psycho-social well-being, including the risk of causing an actual mental health disorder, gambling disorder [1, 2]. Problem gambling is a public health issue affecting people across the globe [3]. Risk factors include male gender, younger age, and poor mental health, but also a more difficult socio-economic situation, which is also known to worsen during problematic gambling [4].

In line with the obvious financial issues related to a problematic gambling behavior, a gambling disorder appears to be strongly linked to over-indebtedness [5], affecting the housing situation and socio-economic conditions of affected individuals and their significant others. In addition to the financial issues, research has increasingly focused on health consequences [1]. These include, among others, depressed mood and anxiety [6, 7], as well as an increased risk of suicidal behavior including death from completed suicide [8]. In the clinical setting, personality disorders appear to be common, and clinical data indicate that suicidal behavior may be particularly common in individuals with a comorbid psychiatric disorder in addition to a gambling disorder [9].

Also, while the focus of gambling-related research is more typically oriented towards mental health, there is also growing evidence that individuals with a gambling disorder face more physical health issues than in the general population, including higher rates of neurological, respiratory, endocrine and digestive diseases [10]. In the setting studied here, a national register study suggested a link between gambling disorder and both cardio-vascular and endocrine disease, as well as a risk increase for problem gambling after bariatric surgery for obesity [11, 12]. Cardio-vascular effects from the gambling situation as such have been suggested [13], but there also have been descriptions of more hazardous lifestyle habits in individuals with gambling problems [14].

However, many barriers have been described against help-seeking for gambling problems [15], and treatment seeking for gambling disorder is generally low; in fact, a majority of individuals with a manifest gambling disorder never seek treatment, and in individuals with an emerging problem or less severe consequences, treatment seeking is rare [16]. Treatment data in health care settings is likely less available for this non-substance-related condition than for substance use disorders, where medications or other medical examinations or interventions are likely more common. Detection and diagnosis of gambling problems may also be less common and more challenging in primary care than for other health conditions [17]. Due to stigma, shame, patient misrepresentation, and structural barriers, screening, diagnosis, and treatment uptake for gambling disorder remain limited and need to be actively promoted in many settings [18].

Within the clinical picture of a severe gambling problem, voluntary self-exclusion from gambling is commonly reported [19]. Self-exclusion is a very common harm reduction measure or an action taken by individuals in order to control their gambling behavior for a certain period of time. However, in a clinical context, self-exclusion has been seen to be both commonly applied and commonly breached, through overseas or otherwise irregular gambling patterns in individuals suffering from an addictive gambling pattern. Also, although in relatively small study samples, patients who self-exclude, and in particular those who self-exclude but breach their gambling ban, are more likely to suffer from mental health disorders [19], making it particularly relevant to study this harm reduction tool in relation to mental health comorbidities.

The present study aims to assess the prevalence and correlates of physical and mental health disorders in clinical patients with a gambling disorder, and how their comorbidities relate to different types of gambling behavior, self-exclusion patterns, to demographic and psycho-social characteristics, and to different conduits to gambling disorder treatment (self-referral versus different routes of referral from other units). In particular, the study also aims to assess which other treatment contacts that patients have had prior to seeking treatment, including at the referring unit and other prior health care contacts, enabling the study to describe the pre-treatment health care consumption of patients with a gambling disorder, potentially facilitating an earlier detection of emerging gambling problems in patients seeking health care for other reasons. All analyses will be conducted with the possibility of comparing and separating women and men (according to legal gender as expressed by the official personal identification number).

More specifically, research questions to be analyzed include the following:

- Which physical comorbidities are seen in patients seeking treatment for a gambling disorder, and how do they differ between different routes of referral?
- Which psychiatric comorbidities are seen in patients seeking treatment for a gambling disorder, and how do they differ between different routes of referral?
- How do demographic and socioeconomic factors (such as over-indebtedness, housing and employment status) differ between patients with or without physical comorbidities?
- How common is tobacco use and alcohol-related problems in patients seeking treatment for gambling disorder, and do they differ between different gambling patterns and routes of referral?
- How common is voluntary self-exclusion from gambling and the breaching of such self-exclusion, and how does that vary between groups reporting different gambling types, and across physical and psychiatric comorbidities, age, gender and different socio-economic status?

## Methods/design

### Setting

The present study focuses on a broad spectrum of health issues in patients seeking treatment for gambling disorder, i.e. for the diagnosis coded as F63.0 in the diagnostic system used in the present setting, the International Classification of Diseases, 10th revision, the ICD-10 [20]. It studies 10 years of treatment seeking at a regional, specialized gambling disorder treatment unit within the health care services (in psychiatry) in Sweden. This is a retrospective health care documentation and register study. Included individuals are all patients (estimated to be a total of around 1,200) seeking treatment at a gambling disorder treatment unit in Skåne county, Sweden, from the opening of the facility in December, 2015, through November, 2025, i.e. for a duration of 10 years. The uptake area of this facility includes one administrative health care region (county) with a population of 1.4 million inhabitants. The facility is located in the region’s most urban setting, the city of Malmö, with a population of around 360,000 inhabitants. This unit admits and treats adults, i.e. treatment-seeking individuals aged 18 years or older.

In Sweden, around 2% of the population are believed to suffer from moderate-risk or problem gambling, according to the most recent scientific publication of nationwide survey data on gambling from 2014 [21]. After a history where gambling problems were primarily addressed by voluntary patient and peer support organizations, and rarely by public institutions [22], a change in legislation in 2018 made it mandatory for regional health care settings and for municipal social services to offer support and treatment for gambling disorder [23]. Before that, in parallel with a research initiative in 2015, the first Swedish health care treatment unit for gambling problems had started in the health care organization of Region Skåne, i.e. the unit assessed in the present paper [24]. In parallel with the all-county health care facility described here, social services of all 33 municipalities in the same geographical region share a responsibility of assessment and interventions against gambling disorder as well as for substance use disorders, and they may work along with, or in collaboration with, the health care-oriented treatment unit, or may also treat clients with or without referring to the health care unit. Despite the new legislation, treatment seeking and treatment uptake for gambling disorder likely remain low nationwide, even when considering both the data on social service interventions and medical diagnostic and treatment interventions, combined. Recently, it has been reported that the total number of individuals diagnosed annually for gambling disorder is approximately 1,000, with a similar (and likely overlapping) number reported from social services in the country, thereby representing a small minority of all inhabitants likely to suffer from a gambling problem on a diagnostic level [25].

The treatment unit assessed here has been described in previous scientific literature. In an early follow-up of the first 16 months of this treatment unit, 58% of the patients were reported to suffer from at least one more mental health disorder [24]. Large gender differences have been described with respect to types of gambling and mental health comorbidity [19, 24], and rates of self-exclusion and the risk of breaching one’s self-exclusion has been documented in different papers originating from the facility [19, 26, 27].

### Data collection

Study data will be extracted from the clinical documentation, i.e. through manual reading of patient records, as well as through a register search in health care registers from the present region (Region Skåne health care databases).

The variables included in the study will describe socio-demographic characteristics (legal gender, age, housing and working conditions, as well as socio-economic characteristics), co-morbid physical and psychiatric disorders (following the International Classification of Diseases, tenth revision, World Health Organization, 2004 [20]), and categorized in sub-categories in line with procedures from prior work in this area [28], prescribed medications of all types, routes of referral, emergency psychiatric contacts, tobacco use and identified alcohol or drug problems requiring treatment, as well as gambling-specific data. The latter will include history of self-exclusion from gambling and each of the types of gambling reported by the patients, and will also cover all gambling types known to occur in the clinical setting [19] as well as in survey studies from the present setting [29]. In addition to traditional gambling types, this will also include the minority of patients reporting a gambling behavior represented by gambling for money or values of a monetary value, within a videogame context, a type of gambling recognized to present a novel but relevant challenge in the context of a gambling disorder [30, 31]. A detailed list of included study items can be found in Table 1.

**Table 1:** Variables to be included in the study, derived from either manual reading of health care documentation, or from the regional health care register data extraction

Variable	Manual reading of patient records.	Region Skåne health care databases (regional health care registers).	Response options/units
Gender (legal)	x	x	Male (legal), female (legal).
Age	x	x	Years
Information about alcohol problems requiring treatment	x		Yes, no, missing
Tobacco use	x		Smoked tobacco, smokeless tobacco, both, none of the above
Gambling type(s) reported by the patient (any one reported to be used)	x		Several options possible: Online casino, online sports betting, land-based sports betting, online horse race betting, land-based horse race betting, land-based electronic gambling machines, online bingo, land-based bingo, online poker, land-based card games, gambling within videogames, cryptocurrency/stock exchange/financial instrument speculation, other, missing.
Gambling type(s) reported by the patient (perceived by patient as problematic)	x		Several options possible: Online casino, online sports betting, land-based sports betting, online horse race betting, land-based horse race betting, land-based electronic gambling machines, online bingo, land-based bingo, online poker, land-based card games, gambling within videogames, cryptocurrency/stock exchange/financial instrument speculation, other, missing.
History of self-exclusion on national self-exclusion service (the *Spelpaus* service).	x		Yes, no, missing.
Gambling during ongoing *Spelpaus* self-exclusion (‘breaching’ of self-exclusion).	x		Yes, no, missing, not applicable (the latter in case of not reporting ever being self-excluded).
Housing conditions	x		Private house or apartment, living in somebody else’s private housing, housing provided by social services, homeless, other, missing.
Socio-economic situation	x		Over-indebtedness, contacts with enforcement agency for over-indebtedness, current or previous debt settlement, missing.
Working conditions	x		Unemployed, temporary employment, employment, other, missing.
Co-morbid physical and mental health disorders (from gambling disorder unit, other psychiatric units, specialized physical health care, or primary care)		x	All diagnostic categories, see Table 2 for full description.
Prescribed medications (from all units)		x	See Table 3 for full description.
Emergency hospital visits		x	All visits (if any) to psychiatric emergency units, and start and end (admittance and discharge) of all psychiatric in-patient treatment episodes (if any)
Referring units (unit referring the patient to the gambling disorder unit)	x		Primary care, occupational health care, other psychiatry, social services, somatic health care, other, self-referred.

**Table 2:** Structure of physical and psychiatric diagnoses for data extraction from health care register database. Diagnoses according to the International Classification of Diseases, ICD-10

ICD-10 code (s)	Disorders
AB	Certain infectious and parasitic diseases
CD	Neoplasms, diseases of the blood, and diseases involving the immune mechanism
E	Endocrine, nutritional and metabolic diseases
G	Diseases of the nervous system
H	Diseases of the eye and the ear
I	Diseases of the circulatory system
J	Diseases of the respiratory system
K	Diseases of the digestive system
L	Diseases of the skin
M	Diseases of the musculoskeletal system
N	Diseases of the genito-urinary system
O	Pregnancy, childbirth and the puerperium
Q	Congenital malformations
R	Symptoms, signs and findings, not elsewhere classified
ST	Injury, poisoning, and other consequences of external causes
V0-X59	Accidents
X40-X49	Accidental poisoning
X60-X84	Intentional self-harm
X85-Y09, Y35-Y36	Assault, legal intervention or operation of war
Y10-Y34	Events of undetermined intent
Y40-Y98	Other external causes
F00-F09	Organic, including symptomatic, mental disorders
F10	Mental and behavioral disorders due to use of alcohol
F11-F19	Mental and behavioral disorders due to use of drugs
F20-F29	Schizophrenia, schizotypal and delusional disorders
F32-F33	Depressive disorders
F30-F31	Bipolar disorders
F34-F39	Other mood disorders
F40-F48	Neurotic, stress-related and somatoform disorders
F50	Eating disorders
F52	Sexual disorders
F60-F62	Personality disorders
F63	Impulse control disorders (excluding gambling disorder, F63.0)
F64-F69	Other disorders of personality
F70-F79	Mental retardation
F80-F89	Pervasive and specific developmental disorders
F90-F98	Behavioral and emotional disorders with onset usually occurring in childhood and adolescence
F99	Unspecified disorder

**Table 3:** Structure of data extraction from health care register database: prescribed pharmaceuticals according to the Anatomical Therapeutic Chemical (ATC) categorization

Categories of medications	ATC codes
***Psychiatric medications***	
Antidepressive medications	N06A
Analgesics	N02
Antiepileptics and mood stabilizers	N03
Antiparkinsonian medications (including dopamine agonists used for restless legs syndrome)	N04
Sedative and anxiolytic medications, and antipsychotics	N05, R06AD01 (alimemazine), R06AD02 (prometazine), R06AD52 (promethazine combinations)
Psychoanaleptics	N06
Anti-addictive medications	N07B
***Cardiometabolic***	
Antithrombotic agents (antiplatelets and anticoagulants)	B01
Cardiac therapy (arrhythmia, heart failure, nitrates)	C01
Antihypertensives, diuretics, beta blockers, calcium channel blockers and RAAS agents	C02, C03, C07, C08, C09
Lipid modifying agents	C10
Drugs used in diabetes	A10
***Respiratory***	
Allergy / antihistamines	R06 (excl. R06AD, see psychiatric section)
Drugs for obstructive airway diseases (asthma/COPD)	R03
***Gastrointestinal***	
Drugs for acid-related disorders (PPIs, H2-antagonists)	A02
***Musculoskeletal and inflammation***	
Anti-inflammatory and antirheumatic products (NSAIDs)	M01
***Endocrine***	
Thyroid therapy (hypo- and hyperthyroidism)	H03
Corticosteroids for systemic use	H02
***Infections and immunomodulation***	
Antibacterials for systemic use	J01
Antivirals for systemic use (incl. HIV, hepatitis)	J05
Immunosuppressants (incl. transplant)	L04

The regional health care registers will be complete and do not typically suffer from missing data on primary and secondary diagnoses, or prescribed pharmaceuticals, and all individual data is linked to an identity-specific Swedish social security number, which includes dates of birth (and thereby exact age) as well as a figure indicating legal gender (male of female). For chart review data, items can be missing, such as in circumstances where typical admittance routines have not been adhered to, or where the primary assessments have been discontinued upon patients’ request or due to severe mental distress in the patients (which, however, in clinical experience from the unit, is very rare). In the manual reading of the health care documentation to be included here, items will be recorded as missing if data cannot be found in the documentation. Manual reading will follow a protocol in line with the response options listed in Table 1. Three to four assessors (with clinical experience, licensed physicians, nurses, or social workers familiar with the project’s intentions) are planned to carry out the manual data extraction. Reading of different assessors will overlap; 10% of each assessors’ records will be reviewed independently by another assessor, in order to be able to describe inter-rater reliability. In cases on which assessors do not agree, the files will be assessed together until consensus is reached.

### Statistical analysis

Descriptive statistics will be used to characterize the study population in terms of demographic and psycho-social variables, gambling patterns, comorbidities, and routes of referral. Associations between physical and mental health comorbidities and gambling-related variables, including gambling type, self-exclusion status, and gambling despite self-exclusion, as well as demographic characteristics, psycho-social living conditions, prescribed medications, and referral routes, will be examined using logistic regression analyses, adjusted for age and gender. Any logistic regression used in the analyses will, conservatively, include only items which appear to display significant associations in uncontrolled, binary comparisons (although age and legal gender will be added to all regression analyses in order to correct for potential biases in demographic characteristics, given the large gender differences seen in gambling and the marked difference in age, on a group level, between women and men with a gambling disorder in the present setting [28]). Also, before entering variables into a logistic regression model, a binary correlation matrix will be displayed for all variables to be included, in order to avoid inclusion of variables with a high degree of correlation (*r* > 0.7), and in the case of a too high correlation between one pair of variables, the variable with the strongest association with the outcome measures in uncontrolled analyses will be the one entered. For sub-analyses where several group comparisons are made, for example in the comparisons between women and men, Bonferroni corrections will be applied in order to prevent over-reporting of false positive group differences (type I error).

In order to assess potential changes in treatment seeking and comorbidity patterns over the study period, including changes potentially associated with the 2018 shift in legislation, changes over time across the study period will be assessed using year of treatment entry as a continuous or categorical variable.

Missing data will be reported for each variable, but not replaced. Where more than 20% of responses for a variable are missing, sensitivity analyses will be carried out to evaluate whether this affects the findings. Kappa statistics will be used for the assessment of inter-rater reliability. All analyses will be conducted in SPSS version 29 (IBM Corp., Armonk, NY), and statistical significance will be set at *p* < 0.05.

### Patient and public involvement

The research group has a close collaboration with the national patient and peer support organization [32], which will review the project along the analysis of data and interpretation of scientific conclusions and their implementation. A communication plan for the study is established for the active dissemination of study results to political and authority decision makers, media and stakeholders in health care and social services.

### Study pre-registration

The study was pre-registered at clinicaltrials.gov, with Clinical Trials identifier number NCT07488871, on March 9, 2026.

## Discussion

Given the low degree of treatment seeking in gambling disorder, its shorter history of clinical implementation compared to substance-related disorders, and the relatively recent incorporation of gambling in clinical contexts, there is reason to assess characteristics of the treatment uptake when a new facility opens up specifically for gambling disorder, within an otherwise more substance-oriented clinical addiction setting. After 10 years of running such a unit, important conclusions may be drawn about the population seeking and being referred for treatment, and based on previously known barriers against treatment, the study has potential to highlight circumstances which should have evoked other providers of treatment to screen for or to refer a person for a suspected gambling problem. In addition, given the relatively long time frame of the study and the longitudinal variations seen in the gambling market, the present study can, for example, detect differences over time in the distribution of gambling types, changes after gambling was officially highlighted in Swedish treatment legislation in 2018 [23], changes after the gambling market was legally re-regulated in 2019 [26], and after the outburst of the COVID-19 pandemic [33].

Few settings have access to large-scale register data allowing for clinical data to be run against a large range of other treatment providers within a geographical area. Sweden has a history of such large-scale registers for health care, such as diagnoses and prescribed medications, including for the study of gambling disorder patients [25, 34]. However, the contribution of the present project, in addition to the combining of registers, will be the possibility to manually extract detailed patient record data, such that health care register data can be compared with descriptions of gambling patterns and psycho-social characteristics surrounding the gambling problem and which cannot readily be detected in any automated register source. For this reason, the combination of clinical and register data has the potential to widen the picture of treatment seeking patterns in gambling disorder.

### Limitations

In a routine clinical setting, where patients, in the perspective of a variety of mental health comorbidities and psycho-social functioning, and in some cases a low patient adherence to treatment sessions, the uptake of study variables may not always be complete. However, more detailed socio-economic or gambling-related characteristics are typically not available in large-scale register databases, such that a thorough manual reading of health care documentations is likely to provide the most extensive data source possible for such patient data. This, for example, applies to the specific gambling pattern of patients. In a previous study of a limited part of the time frame to be studied here, the type of gambling involved was missing in 13% of the patient records [19]. However, this was the case predominantly before the introduction of a quality register used in the present clinical setting and which is likely to have improved structured documentation [35], and which is likely to keep the percentage of missing gambling types on a low level.

Another potential study limitation is the fact that indeed, this study of a treatment facility involves only individuals who have chosen to seek treatment, and therefore the study cannot lead to conclusions about individuals with a corresponding degree of gambling problem but who refrain from seeking treatment. It has been suggested that these groups may differ; clients seeking treatment are likely to have a higher degree of problem severity and awareness of their problem or may have a higher degree of motivational pressure from others [36]. Therefore, the present project cannot draw conclusions about characteristics of the population in general with a gambling disorder.

## Data Availability

No datasets were generated or analysed during the current study.
